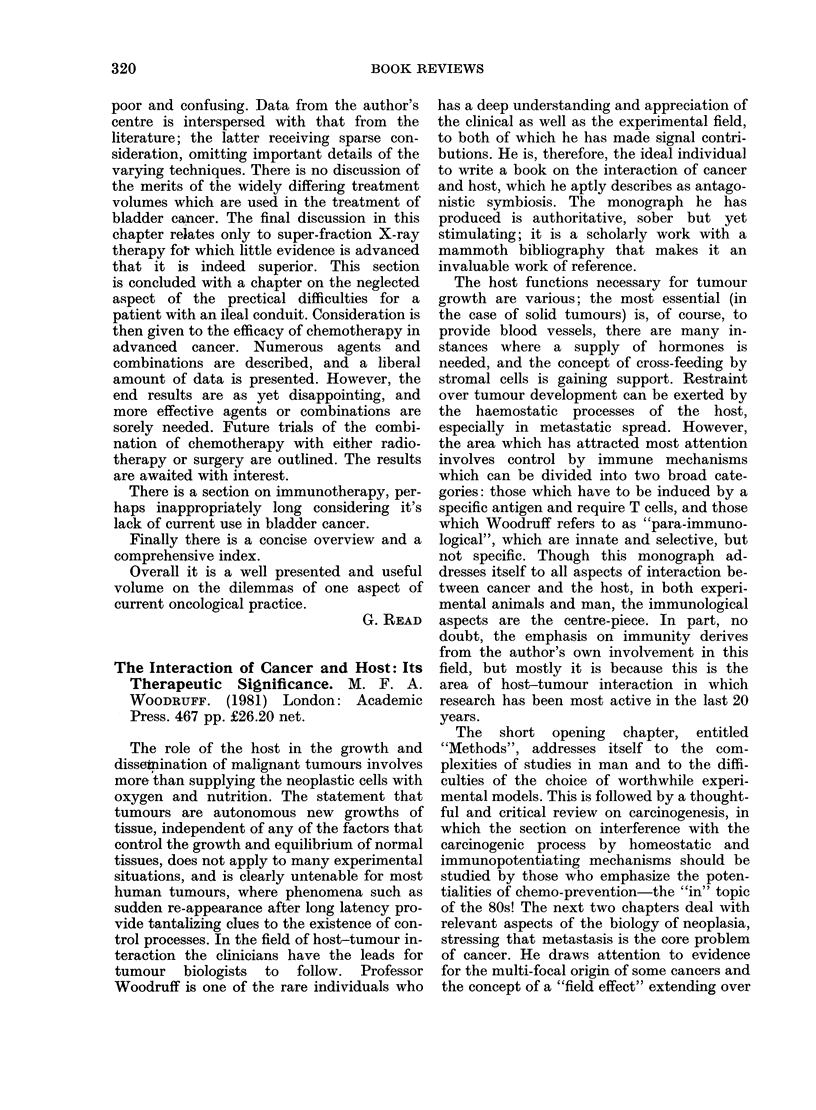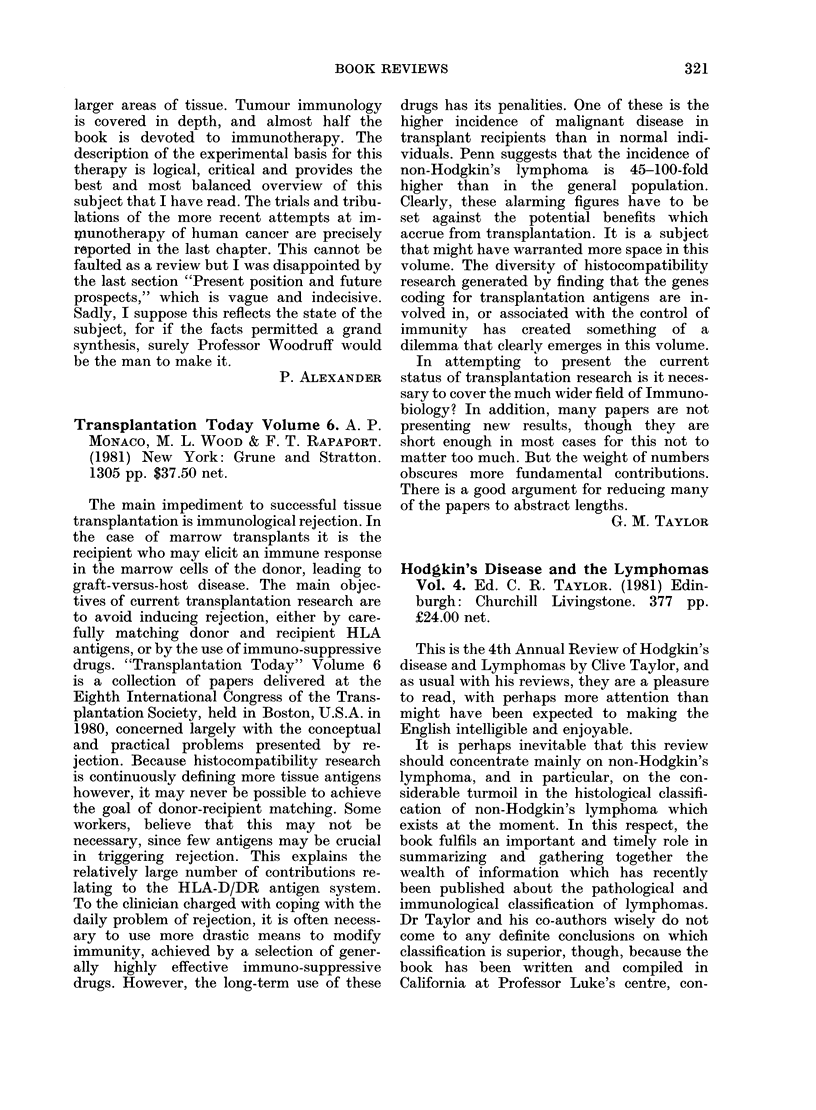# The Interaction of Cancer and Host: Its Therapeutic Significance

**Published:** 1982-02

**Authors:** P. Alexander


**The Interaction of Cancer and Host: Its Therapeutic Significance.** M. F. A. Woodruff. (1981) London: Academic Press. 467 pp. £26.20 net.

The role of the host in the growth and dissemination of malignant tumours involves more than supplying the neoplastic cells with oxygen and nutrition. The statement that tumours are autonomous new growths of tissue, independent of any of the factors that control the growth and equilibrium of normal tissues, does not apply to many experimental situations, and is clearly untenable for most human tumours, where phenomena such as sudden re-appearance after long latency provide tantalizing clues to the existence of control processes. In the field of host–tumour interaction the clinicians have the leads for tumour biologists to follow. Professor Woodruff is one of the rare individuals who has a deep understanding and appreciation of the clinical as well as the experimental field, to both of which he has made signal contributions. He is, therefore, the ideal individual to write a book on the interaction of cancer and host, which he aptly describes as antagonistic symbiosis. The monograph he has produced is authoritative, sober but yet stimulating; it is a scholarly work with a mammoth bibliography that makes it an invaluable work of reference.

The host functions necessary for tumour growth are various; the most essential (in the case of solid tumours) is, of course, to provide blood vessels, there are many instances where a supply of hormones is needed, and the concept of cross-feeding by stromal cells is gaining support. Restraint over tumour development can be exerted by the haemostatic processes of the host, especially in metastatic spread. However, the area which has attracted most attention involves control by immune mechanisms which can be divided into two broad categories: those which have to be induced by a specific antigen and require T cells, and those which Woodruff refers to as "para-immunological", which are innate and selective, but not specific. Though this monograph addresses itself to all aspects of interaction between cancer and the host, in both experimental animals and man, the immunological aspects are the centre-piece. In part, no doubt, the emphasis on immunity derives from the author's own involvement in this field, but mostly it is because this is the area of host–tumour interaction in which research has been most active in the last 20 years.

The short opening chapter, entitled "Methods", addresses itself to the complexities of studies in man and to the difficulties of the choice of worthwhile experimental models. This is followed by a thoughtful and critical review on carcinogenesis, in which the section on interference with the carcinogenic process by homeostatic and immunopotentiating mechanisms should be studied by those who emphasize the potentialities of chemo-prevention—the "in" topic of the 80s! The next two chapters deal with relevant aspects of the biology of neoplasia, stressing that metastasis is the core problem of cancer. He draws attention to evidence for the multi-focal origin of some cancers and the concept of a "field effect" extending over larger areas of tissue. Tumour immunology is covered in depth, and almost half the book is devoted to immunotherapy. The description of the experimental basis for this therapy is logical, critical and provides the best and most balanced overview of this subject that I have read. The trials and tribulations of the more recent attempts at immunotherapy of human cancer are precisely reported in the last chapter. This cannot be faulted as a review but I was disappointed by the last section "Present position and future prospects," which is vague and indecisive. Sadly, I suppose this reflects the state of the subject, for if the facts permitted a grand synthesis, surely Professor Woodruff would be the man to make it.

P. ALEXANDER